# Magnetic carboxyl functional nanoporous polymer: synthesis, characterization and its application for methylene blue adsorption

**DOI:** 10.1038/s41598-018-24873-3

**Published:** 2018-04-25

**Authors:** Hongxin Su, Weiwei Li, Yide Han, Ningning Liu

**Affiliations:** 1 College of Chemistry, Chemical Engineering and Environmental Engineering, Liaoning Shihua University, Fushun, 113001 P.R. China; 2 Department of Chemistry, College of Science, Northeastern University, Shenyang, 110819 P.R. China

## Abstract

Magnetic carboxyl functional nanoporous polymer (MCFNP) was chemically fabricated by incorporation of magnetic Fe_3_O_4_ precursor into the carboxyl functional nanoporous polymer (CFNP). The as-synthesized MCFNP was characterized and used as an adsorbent for rapid adsorption removal of methylene blue (MB) from wastewater. Several experimental parameters affecting the adsorption efficiency were investigated including initial pH, adsorbent dosage, initial MB concentration, contact time and temperature. The adsorption behavior of MCFNP displayed that adsorption kinetics and isotherms could be well fitted to the pseudo-second-order and Langmuir models, respectively. The experimental results showed that MCFNP was an effective adsorbent with a maximum adsorption capacity of 57.74 mg g^−1^ for MB at 298 K. The negative free energy (ΔG) and positive enthalpy change (ΔH) confirmed that the adsorption reaction was a spontaneous and endothermic process. In addition, ethanol was used as an effective extractant for the regeneration of MCFNP, and the adsorption efficiency could remain 80% after the ninth regeneration cycle.

## Introduction

Nowadays, industrial wastewater treatment is attracting more and more attention due to water scarcity^1^. With the rapid development of industrialization, a large number of untreated industrial effluents are discharged directly into water source, which has a serious impact on environment and human health. Especially, textile wastewater contains toxic substances like dyes, which can detrimentally affect the ecological balance. Thus, a high efficiency and low cost method for wastewater treatment is still urgently required. To date, adsorption is the most commonly used technology in industrial wastewater treatment. A number of adsorbents, such as active carbons^2,3^, clays^4^, zeolites^5,6^, metal organic frameworks (MOFs)^7,8^, mesoporous silica materials^9,10^, polymers^11–18^ and reduced graphene oxides (RGOs)^19,20^, have been developed to remove inorganic and organic contaminants from aqueous solution. Polymers with high adsorption capacity are expected to be potential candidate for the removal of organic pollution from wastewater^11–18^.

Recently, magnetic porous polymer materials have become one of the research focuses since they possess the characteristics of magnetism and porosity^21–25^. These materials as adsorbents can be easily removed from wastewater by using an external magnetic field, indicating great potential in application. Therefore, magnetic porous polymer materials have been widely reported because of their high specific surface area, rich porous structure and high separation efficiency. For example, Huang *et al*. fabricated magnetic porous organic polymer composites with high adsorption capacity toward methylene blue^21^. Chen *et al*. demonstrated that magnetically recoverable cross-linked polyethylenimine possessed excellent adsorption capacity for the removal of organic dyes^22^. Sun *et al*. developed xylan/poly (acrylic acid) magnetic nanocomposites as adsorbents for the adsorption removal of methylene blue^23^. Yao *et al*. reported that porous magnetic polyacrylamide microspheres showed high adsorption capacities for organic dyes^24^. Thus, developing a novel magnetic porous polymer adsorbent is a promising method for water purification.

In this study, magnetic carboxyl functional nanoporous polymer (MCFNP) was fabricated via the chemical method based on the combination of carboxyl functional nanoporous polymer (CFNP) and Fe_3_O_4_ precursor. In order to investigate the adsorption behavior of as-synthesized MCFNP for methylene blue (MB) in detail, the effects of initial pH, adsorbent dosage, initial MB concentration, contact time and temperature were discussed. Adsorption isothermal models, kinetics characteristics and thermodynamic parameters were also analyzed.

## Results

### Characterization of MCFNP

**Figure 1:**
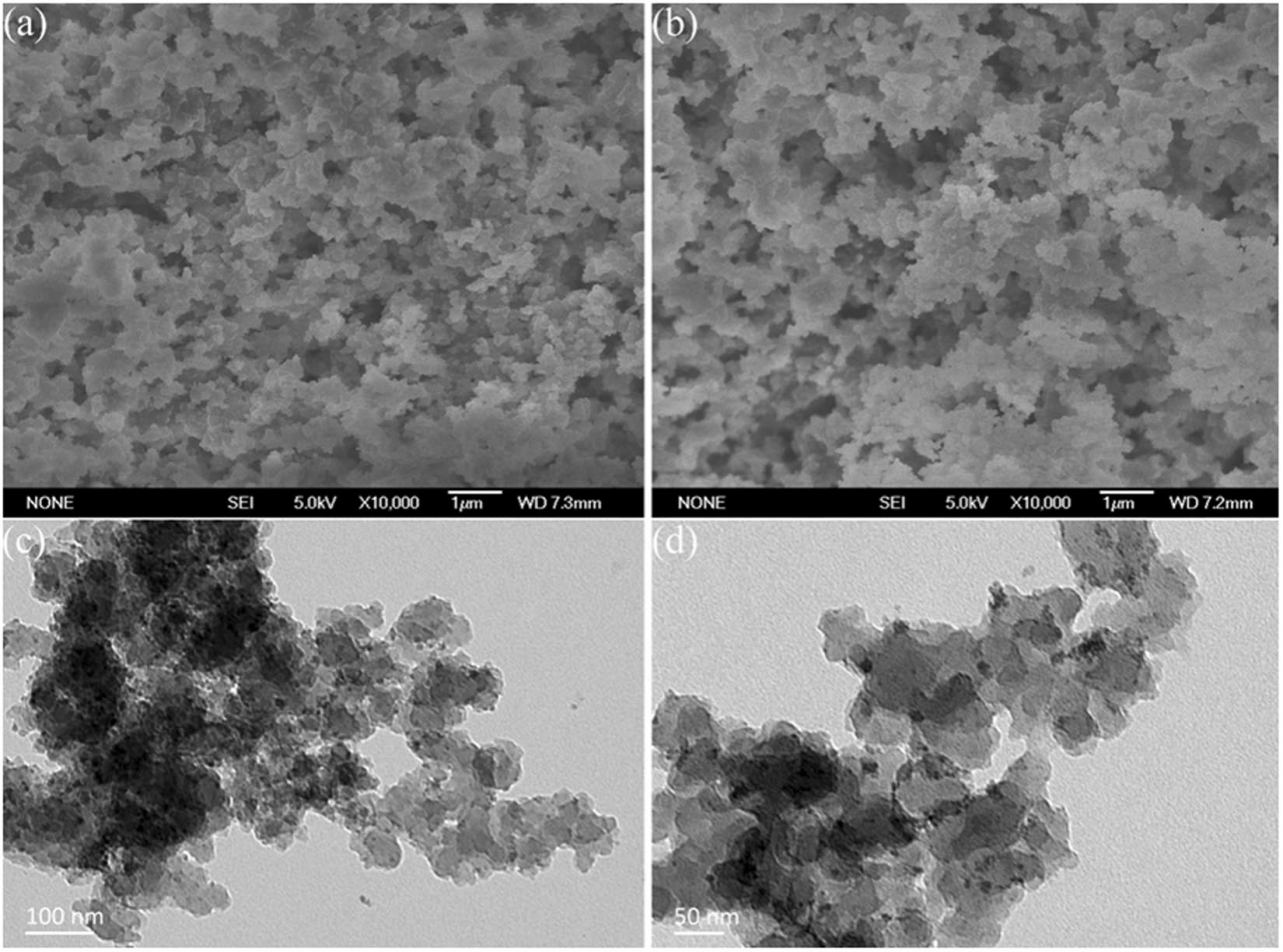
(**a**) and (**b**) SEM images of MCFNP and CFNP, (**c**) and (**d**) TEM images of MCFNP.

Figure 1a,b show the SEM images of MCFNP and CFNP, respectively. It can be clearly observed that MCFNP and CFNP have rough surfaces and almost the same morphology. Figure 1c,d show the TEM images of MCFNP. These images display abundant nanoporous and well-dispersed Fe_3_O_4_ particles.

XPS was used to further analyze the composition of MCFNP surface (Supplementary Fig. S1). It can be seen that the elemental contents of the surface are C and O. For iron ions, the binding energy of Fe 2p^3^ at 710 eV cannot be obviously achieved, which results from its low content on the surface.

**Figure 2:**
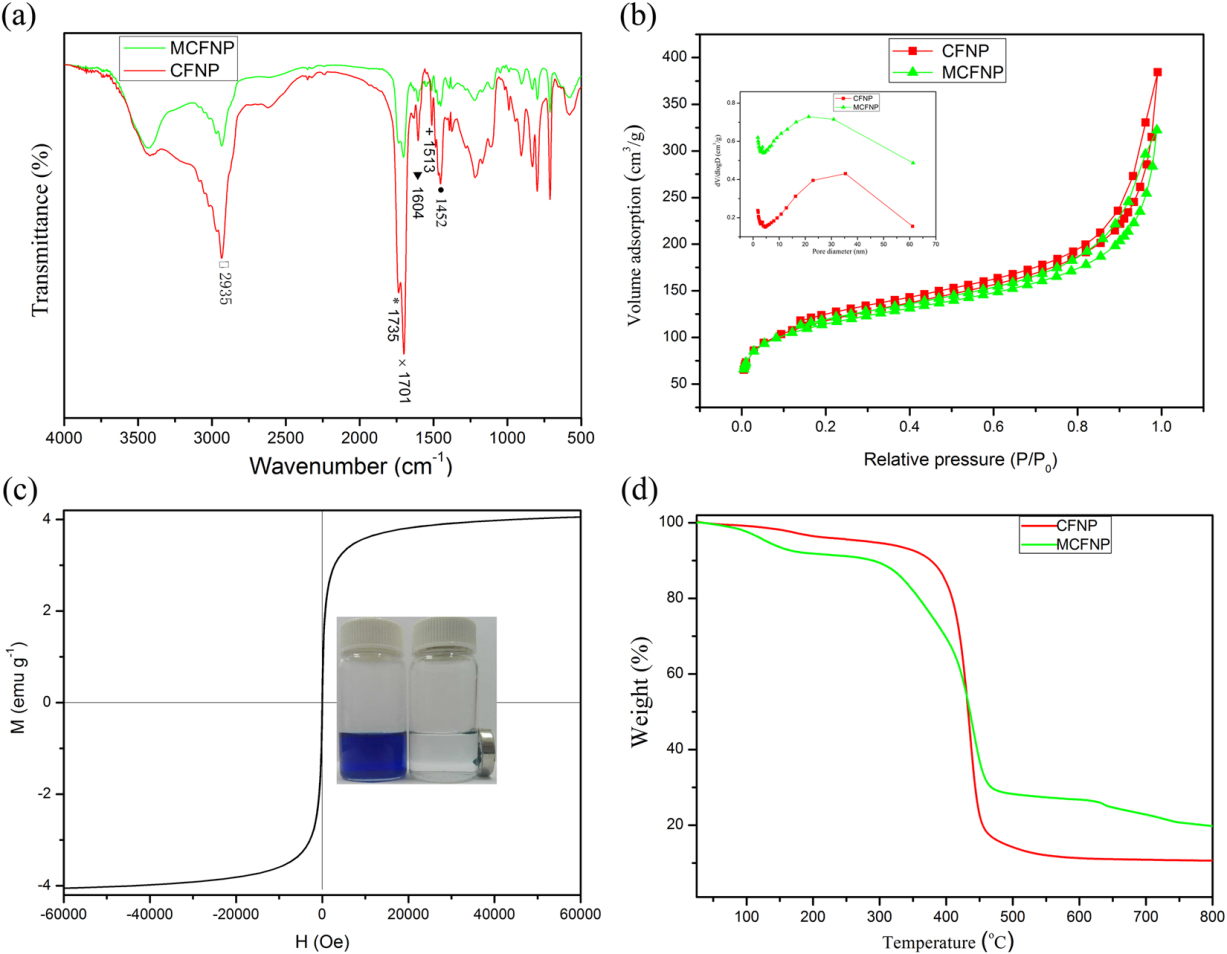
(**a**) FTIR spectra of MCFNP and CFNP, (**b**) N_2_ adsorption-desorption isotherms and pore size distributions of MCFNP and CFNP (Pore size distribution of MCFNP has been offset by 0.4 cm^3^ g^−1^), (**c**) magnetization curve of MCFNP at room temperature and (**d**) TG analyses of MCFNP and CFNP.

The Fourier transform infrared (FTIR) spectra of MCFNP and CFNP are shown in Fig. 2a. As expected, the peaks at 1513, 1486 and 1452 cm^−1^ are attributed to the characteristic stretching vibrations of benzene ring. The band at 3400–3500 cm^−1^ indicates the presence of hydroxyl groups. The peak at 2935 cm^−1^ is ascribed to the C-H bond stretching vibration of methylene. The peak appearing at 1604 cm^−1^ corresponds to the C=C bond stretching vibration. The peaks at 1735 and 1701 cm^−1^ can be assigned to the C=O stretching mode of carboxylic acid groups. It is clear that there exist a high content of carboxyl groups in MCFNP and CFNP, which might be beneficial to MB adsorption.

Figure 2b shows nitrogen adsorption-desorption isotherms and corresponding pore size distributions of MCFNP and CFNP. A typical type IV sorption behaviour with H1-type hysteresis loop is observed in the two samples, suggesting the existence of nanoporous structure. The BET specific surface areas of MCFNP and CFNP are 380 and 394 m^2^ g^−1^, respectively. The deceasing specific surface area of MCFNP is believed that the incorporation of Fe_3_O_4_ might block the pore of MCFNP. The pore size distributions of MCFNP and CFNP are in the range of 20 nm to 40 nm, and the pore volumes are 0.50 and 0.59 cm^3^ g^−1^, respectively. Besides, the fact that MCFNP possesses relatively high specific surface area and large pore size could be beneficial to its adsorption performance.

Magnetic property of MCFNP was measured at room temperature and its magnetic hysteresis curve is shown in Fig. 2c. It can be seen that the sample exhibits paramagnetic character and its saturation magnetization is 4.08 emu g^−1^. This confirms that MCFNP can be rapidly separated under an external magnetic field within 10 min, indicating that the adsorbent has a good application prospect in wastewater treatment.

The thermal stabilities of MCFNP and CFNP are displayed in Fig. 2d. The first weight losses of MCFNP and CFNP before 300 °C are about 11% and 6%, respectively, which is attributed to the residue solvent in the polymers. The second weight loss of 61% and 80% occurred between 300 °C and 500 °C, which might be caused by the decomposition of the polymers. These results mean that the polymers have good thermal stability before 300 °C.

### Effect of initial pH

**Figure 3:**
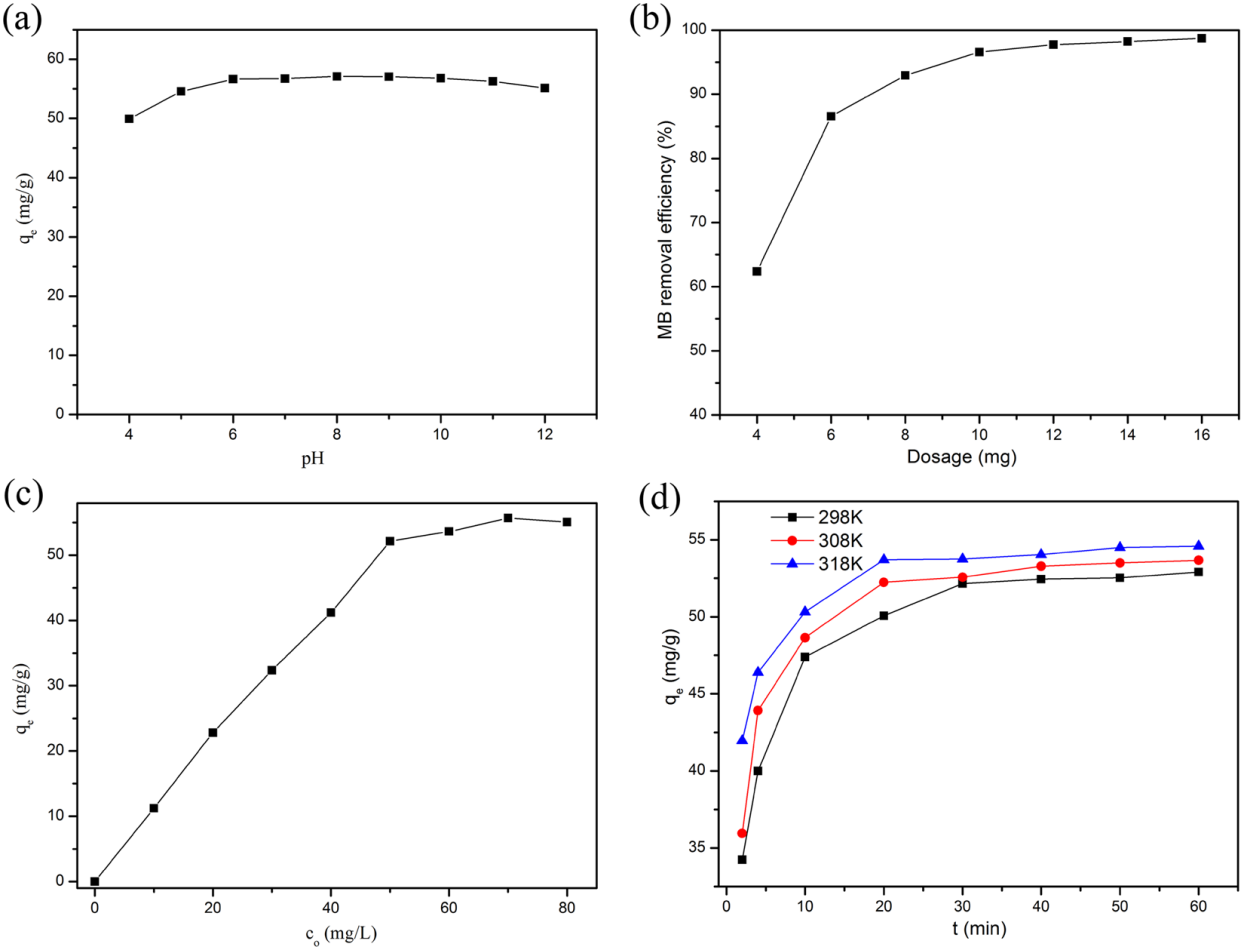
(**a**) Effect of initial pH on the adsorption of MB onto MCFNP, (**b**) effect of adsorbent dosage on adsorptive removal of MB, (**c**) effect of different initial concentrations on the adsorption of MB onto MCFNP and (**d**) Effect of contact time on the adsorption of MB onto MCFNP at various temperatures.

The pH value of MB solution is an important parameter which influences the adsorption capacity of the adsorbent. MB adsorption onto MCFNP in various pH solutions was studied at 298 K. In this work, 6 mg of MCFNP was mixed with 7 mL of MB solution (50 mg L^−1^). The effect of pH is shown in Fig. 3a. The result exhibits that the adsorption capacity increases when the pH values of solution are in the range from 4 to 6. This result might be caused by the competition between H^+^ ions and dye cations for the surface available sites at lower pH. However, when the pH values are 6–12, the adsorption capacity is almost constant. This means that the pH values have no impact on the adsorption efficiency at higher pH. Therefore, the following adsorption test was carried out at pH = 6.

### Effect of adsorbent dosage

The influence of different adsorbent dosages (4, 6, 8, 10, 12, 14 and 16 mg) on the removal of MB (50 mg L^−1^, 7 mL) was investigated at 298 K. The experimental result is given in Fig. 3b. The MB removal efficiency increases with the increasing MCFNP dosage and the maximum removal efficiency is achieved when the dosage of adsorbent is 10 mg. The increase in MB removal efficiency might be attributed to the increase of specific surface area and available adsorption sites of MCFNP.

### Effect of initial MB concentration

To estimate the effect of different initial MB concentrations, 6 mg of MCFNP was mixed with 7 mL of MB solution with different initial concentrations (10–80 mg L^−1^) at 298 K. Figure 3c shows the adsorption capacity of MCFNP in different initial MB concentration solutions. The adsorption capacity of MCFNP is found to increase with increasing initial MB concentration and attain the maximum at 50 mg L^−1^. When the initial MB concentration further increases, the adsorption capacity of MCFNP has almost no change. This indicates that the available adsorption sites of adsorbent could not be occupied by more MB molecules.

### Effects of contact time and temperature

Six milligram of MCFNP was added into 7 mL MB solution of 50 mg L^−1^ at different temperatures (298 K, 308 K and 318 K), then the concentration of MB solution was measured after different time intervals (2–60 min). From Fig. 3d, it can be easily seen that the equilibriums were achieved within 30 min at various temperatures. Before the contact time of 30 min, the adsorption capacity of MCFNP increases with increasing contact time at different temperatures. At 298 K, the equilibrium adsorption amount was obtained at 30 min, 52.16 mg g^−1^. At 308 K and 318 K, the equilibrium adsorption amounts were also obtained, 52.58 and 53.75 mg g^−1^, respectively. Notably, the adsorption capacity of MCFNP at 318 K is higher than that at 298 K and 308 K, which indicates that high temperature is in favour of the occurrence of MB adsorption. This result suggests that temperature is an important parameter for the MB adsorption onto MCFNP.

### Adsorption isotherms

The equilibrium adsorption isotherms have been investigated in detail because they can offer information about the capacities, surface properties and the degree of affinity of adsorbents. Most adsorption isotherms models, including the Langmuir, the Freundlich, the Dubinin-Radushkevich and the Temkin models are applied to fit the equilibrium data. The Langmuir and the Freundlich models are the most common isotherms for describing the adsorption behavior of adsorbent. Thus, in this paper, the Langmuir and the Freundlich models were chosen to fit the equilibrium data.

**Figure 4:**
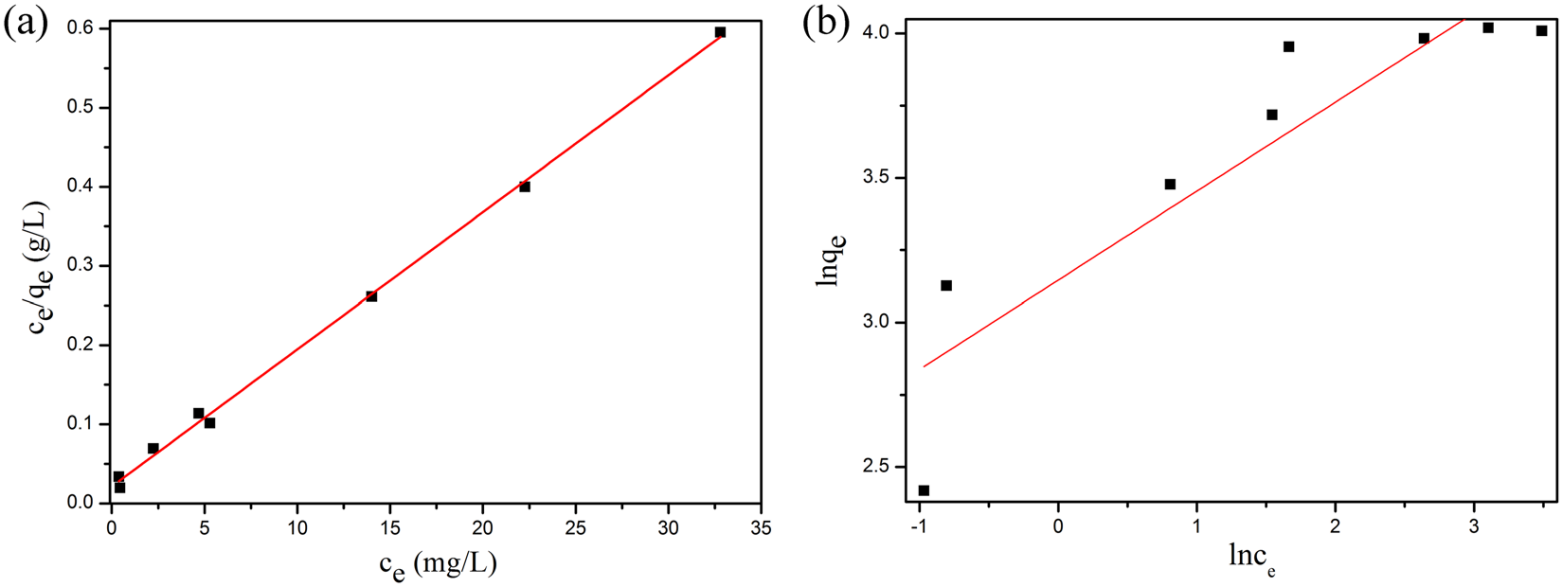
(**a**) Langmuir and (**b**) Freundlich isotherm models for MB adsorption onto MCFNP.

The Langmuir isotherm assumes that monolayer adsorption occurs onto a homogeneous surface and every adsorption site is equivalent and identical; whereas the Freundlich isotherm is an empirical equation assuming that multilayer adsorption process occurs on the heterogeneous surface. Their linear isotherms can be described as follows:

1 $$\frac{{c}_{e}}{{q}_{e}}=\frac{{c}_{e}}{{q}_{m}}+\frac{1}{{q}_{m}{K}_{L}}$$

2 $$\mathrm{ln}\,{q}_{e}=\,\mathrm{ln}\,{K}_{F}+\frac{1}{n}\,\mathrm{ln}\,{c}_{e}$$

where *c*_e_ and *q*_e_ are the concentration of adsorbate (mg L^−1^) and the adsorption capacity of the adsorbent (mg g^−1^) at equilibrium, respectively. *q*_m_ is the maximum adsorption capacity (mg g^−1^), K_L_ is the Langmuir adsorption constant (L mg^−1^), K_F_ and n are Freundlich constants. The Langmuir and the Freundlich models for MB adsorption onto MCFNP are shown in Fig. 4. The isotherm constants for MCFNP were obtained from these two isotherms and their values are listed in Table 1. In Table 1, it is clearly observed that correlation coefficient of the Langmuir isotherm (R^2^ = 0.997) is higher than that of the Freundlich isotherm (R^2^ = 0.797), indicating that experimental data follow the Langmuir isotherm rather than the Freundlich isotherm. According to the Langmuir isotherm, the maximum adsorption capacity for MB onto MCFNP is 57.74 mg g^−1^ at 298 K, which is higher than that of some of the inorganic adsorbents^26^. Therefore, the adsorption behaviour of MB onto MCFNP follows the Langmuir isotherm, indicating the occurrence of monolayer adsorption.

**Table 1:** Isotherm parameters for MB adsorption onto MCFNP at 298 K.

Langmuir model			Freundlich model		
*q*_m_ (mg g^−1^)	K_L_ (mg g^−1^)	R^2^	n	K_F_ (mg g^−1^)	R^2^
57.74	0.80	0.99776	3.25	23.25	0.79755

The separation factor R_L_ based on the Langmuir isotherm is applied to evaluate the favorability of an adsorption system. It can be defined as follows:

3 $${R}_{L}=\frac{1}{1+{K}_{L}{{\rm{c}}}_{0}}$$

where K_L_ is the Langmuir constant and *c*_0_ is the initial concentration of MB solution. The values of R_L_ are in the range of 0 to 1, indicating that adsorption behaviour is favorable. In this work, the values of R_L_ are found to be in the range of 0 to 0.11 (Supplementary Fig. S2), confirming that the MB adsorption onto MCFNP is favorable.

### Adsorption kinetics

Adsorption kinetics is thoroughly explored, since it can provide important information on the adsorption rate and mechanism. To design the appropriate adsorption systems, two well-known kinetics models, pseudo-first-order and pseudo-second-order rate equations are analyzed. The pseudo-first-order and pseudo-second-order rate equations can be expressed as follows:

4 $$\mathrm{ln}({q}_{e}-{q}_{t})=\,\mathrm{ln}\,{q}_{e}-{k}_{1}t$$

5 $$\frac{t}{{q}_{t}}=(\frac{1}{{k}_{2}{{q}_{e}}^{2}})+\frac{t}{{q}_{e}}$$

where *q*_e_ and *q*_t_ (mg g^−1^) are the amounts of adsorbate onto adsorbent at equilibrium and at any time *t* (min), respectively. k_1_ and k_2_ are the rate constants of the pseudo-first-order model and pseudo-second-order model, respectively.

**Figure 5:**
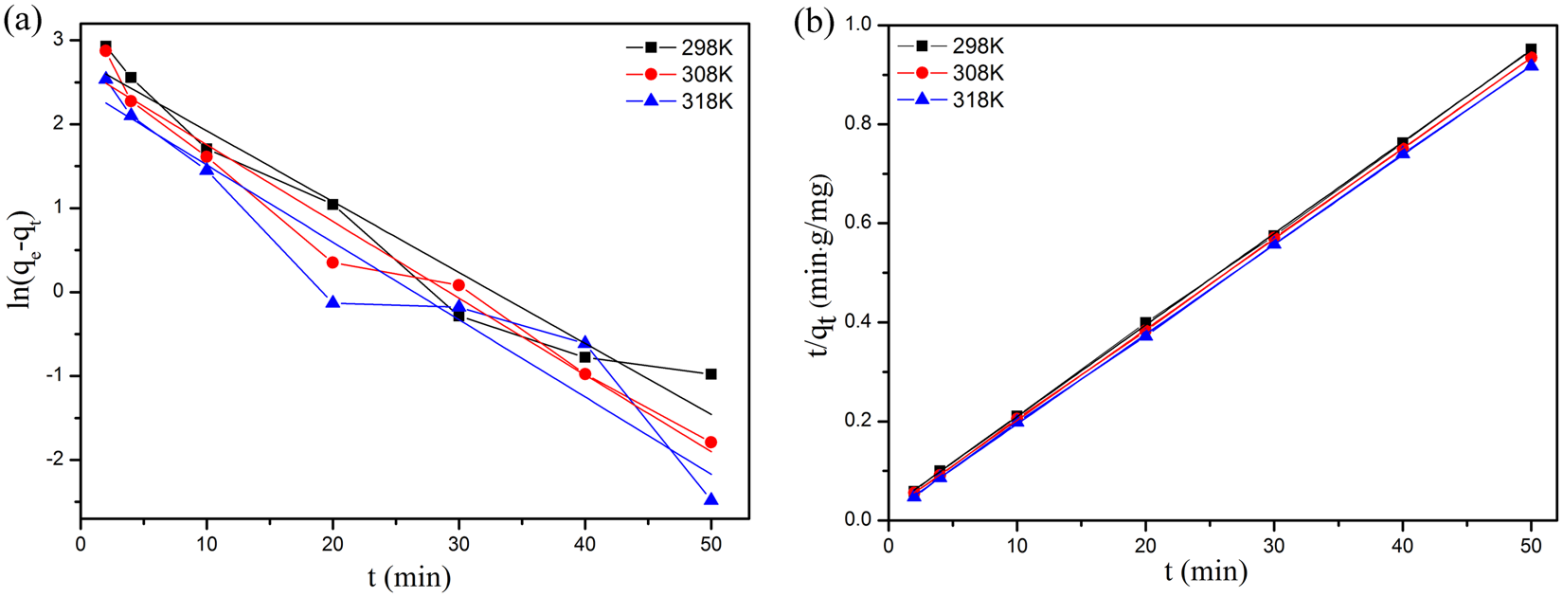
The plots of (**a**) the pseudo-first-order and (**b**) the pseudo-second-order kinetic models for MB adsorption onto MCFNP.

The pseudo-first-order and pseudo-second-order kinetics for the adsorption of MB onto MCFNP at different temperatures are shown in Fig. 5. The kinetics parameters, such as k_1_, k_2_ and *q*_e_, were calculated from linear relationship and listed in Table 2. It is found that correlation coefficients of the pseudo-second-order are higher than that of the pseudo-first-order at different temperatures. This means that the adsorption behaviour of MB onto MCFNP follows the pseudo-second-order model.

**Table 2:** Kinetic parameters of MB adsorption onto MCFNP.

*T* (K)	*q*_e.exp_ (mg g^−1^)	Pseudo-first order model			Pseudo-second order model		
		*q*_e.c_ (mg g^−1^)	k_1_ (min^−1^)	R^2^	*q*_e.c_ (mg g^−1^)	k_2_ (mg g^−1^ min^−1^)	R^2^
298	52.91	15.92	0.08446	0.94527	54.05	0.01391	0.99989
308	53.66	14.44	0.09142	0.96927	54.61	0.01736	0.99996
318	54.58	11.45	0.09219	0.92623	55.22	0.02403	0.99993

### Adsorption thermodynamics

Gibbs free energy change (ΔG), enthalpy change (ΔH) and entropy change (ΔS) are crucial thermodynamic parameters for the investigation of adsorption mechanism. Thus, they are obtained from the following equations:

6 $${K}_{L}=\frac{{q}_{e}}{{c}_{e}}$$

7 $${\rm{\Delta }}G=-\,RT\,\mathrm{ln}\,{K}_{L}$$

8 $$\mathrm{ln}\,{K}_{L}=-\,\frac{{\rm{\Delta }}H}{RT}+\frac{{\rm{\Delta }}S}{R}$$

where *q*_e_ (mg g^−1^) and *c*_e_ (mg L^−1^) are the adsorption capacity of the adsorbent and the concentration of MB in solution at equilibrium, respectively. K_L_ is the Langmuir adsorption constant (L g^−1^). R is the gas constant (8.314 J mol^−1^ K^−1^) and *T* is the solution temperature (K).

Thermodynamic parameters (ΔG, ΔH and ΔS) were determined from the slope and intercept of ln K_L_ versus 1/*T*. The parameters at different temperatures are listed in Table 3. The negative values of ΔG confirm the spontaneous nature and the feasibility of the adsorption process. The ΔG values decrease with increasing temperature, indicating that the adsorption process is more favorable at high temperature. The positive ΔH value means that the adsorption behaviour of MB onto MCFN is endothermic. The ΔS value of MB adsorption onto MCFNP is 72.80 J mol^−1^ K^−1^. The positive ΔS value confirms that there is an increase in the degree of freedom of MB onto MCFNP. Generally, the adsorption of gas onto solid is a process of decreasing entropy because of gas molecules from disordered to ordered state. In the case of MB adsorption onto MCFNP, it is likely that the adsorbed MB molecules on the surface of MCFNP are chaotic. These thermodynamic parameters indicate that MCFNP can be used as a high-efficiency adsorbent to remove MB from wastewater.

**Table 3:** Thermodynamic parameters of MB adsorption onto MCFNP.

*T* (K)	ΔH^θ^ (kJ mol^−1^)	ΔS^θ^ (J mol^−1^ K^−1^)	ΔG^θ^ (kJ mol^−1^)
298	15.70	72.80	−5.992
308			−6.720
318			−7.448

### Regeneration

The cost of adsorbent is a significant parameter for wastewater treatment application. For an adsorbent, recycle is taken as one of the most effective ways to reduce cost. MCFNP can be easily separated from wastewater in the presence of an external magnetic field. And then, the recovery of the spent MCFNP can be achieved by soaking in ethanol for 1 h. Supplementary Fig. S3 shows removal efficiencies of MB on MCFNP after different regeneration cycles, which demonstrates that MCFNP still keeps high MB removal efficiency (80%) after 9 cycles. The results indicate that MCFNP possesses efficient adsorption property, easy separation and good recycling performance for the removal of MB from wastewater. Thus, MCFNP could become a promising adsorbent in the large-scale commercial applications.

## Conclusions

In summary, a MCFNP was successfully prepared by incorporation of magnetic Fe_3_O_4_ precursor into the CFMP which was made from divinylbenzene (DVB) and methacrylic acid (MA). The MCFNP with high surface area and rich porous structure possessed excellent adsorption capacity for MB and good magnetic separation ability. The experimental results showed that MCFNP was an effective adsorbent with a maximum adsorption capacity of 57.74 mg g^−1^ for MB at 298 K. The adsorption behaviour of MCFNP displayed that adsorption kinetics and isotherms could be well fitted to the pseudo-second-order and Langmuir models, respectively. The negative free energy (ΔG) and positive enthalpy change (ΔH) confirmed that the adsorption reaction was a spontaneous and endothermic process. Most importantly, the MCFNP material could be recycled at least nine times with high removal efficiency (80%) as adsorbent for the removal of MB, indicating its potential application prospect.

## Material and Methods

### Materials

Divinylbenzene (DVB) and methacrylic acid (MA) were obtained from Sigma-Aldrich. Ethyl acetate (EtoAc), FeCl_3_·6H_2_O, FeSO_4_·6H_2_O, glycol, NaOH, HCl and MB were supplied by Tianjin Bodi Chemical Co., Ltd., China. 2,2′-azobis(2-methylpropionitrile) (AIBN) was purchased from Shanghai No.4 Reagent & H.V. Chemical Co., Ltd., China. All of these chemicals were of analytical grade and used as received.

### Preparation of CFNP and MCFNP

Typically, 2 g of DVB was dispersed in EtoAc solvent (30 mL), and then 0.07 g of AIBN and 1 g of MA were added into the solution with stirring. After stirring at room temperature for 4 h, the resulting mixture was transferred into a Teflon-lined stainless steel autoclave and heated at 100 °C for 24 h. The dried CFNP was obtained after evaporation of solvent at room temperature.

Two gram of prepared CFNP was dispersed into 40 mL of glycol under vigorous agitation. And then, 1.082 g of FeCl_3_·6H_2_O and 1.036 g of FeSO_4_·6H_2_O pre-dissolved in 10 mL of deionized water were added. After heating at 80 °C for 30 min, 15 mL of NaOH (1.56 mol L^−1^) was added dropwise into the reaction mixture and stirred constantly for another 1.5 h. The sample MCFNP was separated by filtration, washed with distilled water and ethanol, and dried in a vacuum oven at 50 °C (Supplementary Fig. S4).

### Characterization

The size and morphology of the samples were observed by scanning electron microscopy (SEM, ZEISS, Germany) and transmission electron microscope (TEM, Tecnai G220, America), respectively. X-ray photoelectron spectroscopy (XPS) analysis was carried out on an AXIS Ultra. FTIR spectra were obtained over the range 4000-500 cm^−1^ with a Bruker 66 V FTIR spectrometer. Nitrogen adsorption-desorption isotherms were analyzed on a Micromeritics ASAP 2020 M system. The magnetic property was recorded by using a vibrating sample magnetometer (VSM, BHV-55, Japan). Thermo-gravimetric (TG) analyses were performed on a Mettler Toledo TGA2 (LF). The MB concentration and the amount of MB adsorbed onto adsorbents were determinate by using a UV-vis spectrophotometer (TU-1901, Beijing Purkinje General Instrument Co., Ltd. China) at 664 nm.

### Adsorption and desorption test

Six milligram of MCFNP and 7 mL MB solution of 50 mg L^−1^ were placed in a 10 mL centrifuge tube. The mixture was shaken on a constant shaking incubator for 60 min at 298 K, then the adsorbent was separated by a magnet and the absorbance of MB left in the solution was analyzed using a UV-vis spectrophotometer. According to the calibration curve, the concentration of MB was determined. The amount of the adsorbed MB (*q*_e_) and the MB removal efficiency (*R*) was determined according to the following equations (9) and (10), respectively:

9 $${q}_{e}=\frac{({c}_{0}-{c}_{t})V}{m}$$

10 $$R=\frac{({c}_{0}-{c}_{t})}{{c}_{0}}\times 100$$

where *c*_0_ and *c*_t_ are the initial and final concentrations of MB solution, respectively.

To estimate the adsorption performance of different adsorbents (MCFNP, CFNP and Fe_3_O_4_) for MB, adsorption experiments were carried out at 298 K. As can be seen from Supplementary Fig. S5, the adsorption efficiency of MCFNP can reach 90% in 10 min when the concentration of MB is 50 mg L^−1^ and the dosage of adsorbent is 6 mg. This indicates that it is very fast to remove MB from aqueous solution using MCFNP as adsorbent. For comparison, CFNP and Fe_3_O_4_ were also studied for the removal of MB. It is evident that the adsorption efficiency of MCFNP is higher than that of CFNP and Fe_3_O_4_. To investigate this phenomenon, the following experiments were carried out.

Batch adsorption experiments were carried out for investigating the influences of initial pH, adsorbent dosage, initial MB concentration, contact time and temperature for MB adsorption. The pH of initial MB solution was adjusted with 0.1 mol L^−1^ NaOH and 0.1 mol L^−1^ HCl solution in the range from 4 to 12 before the adsorption experiment.

For the desorption experiment, 6 mg of MCFNP was added into 7 mL MB solution of 50 mg L^−1^. The mixture was shaken for 60 min at room temperature. The MB removal efficiency was determined by the calibration curve. The spent adsorbent was re-dispersed in ethanol solution for 60 min, re-collected by a magnet and washed with deionized water. The dried spent adsorbent was used for the following recycle experiment. Each adsorption/desorption experiment was performed three times and only the averages were exhibited in this paper. The accuracy of the experimental results was ± 1%.

## Electronic supplementary material

Supplementary Information
