# Supplementary material for: Magnetic carboxyl functional nanoporous polymer: synthesis, characterization and its application for methylene blue adsorption
Source: Sci Rep. 2018 Apr 25;8:6506. doi: 10.1038/s41598-018-24873-3 (PMC5916890; doi:10.1038/s41598-018-24873-3)
Supplement: Supplementary file 1 — Supplementary Information [file 41598_2018_24873_MOESM1_ESM.pdf]

Supplementary Information

Magnetic carboxyl functional nanoporous polymer: synthesis, characterization and its application for methylene blue adsorption

Hongxin Su[1], Weiwei Li[2], Yide Han[2], Ningning Liu[1,*]

[1]College of Chemistry, Chemical Engineering and Environmental Engineering, Liaoning Shihua University, Fushun, 113001, P.R. China.

[2]Department of Chemistry, College of Science, Northeastern University, Shenyang, 110819, P.R. China.

Correspondence and requests for materials should be addressed to N.L. (email: liuningningf@126.com)

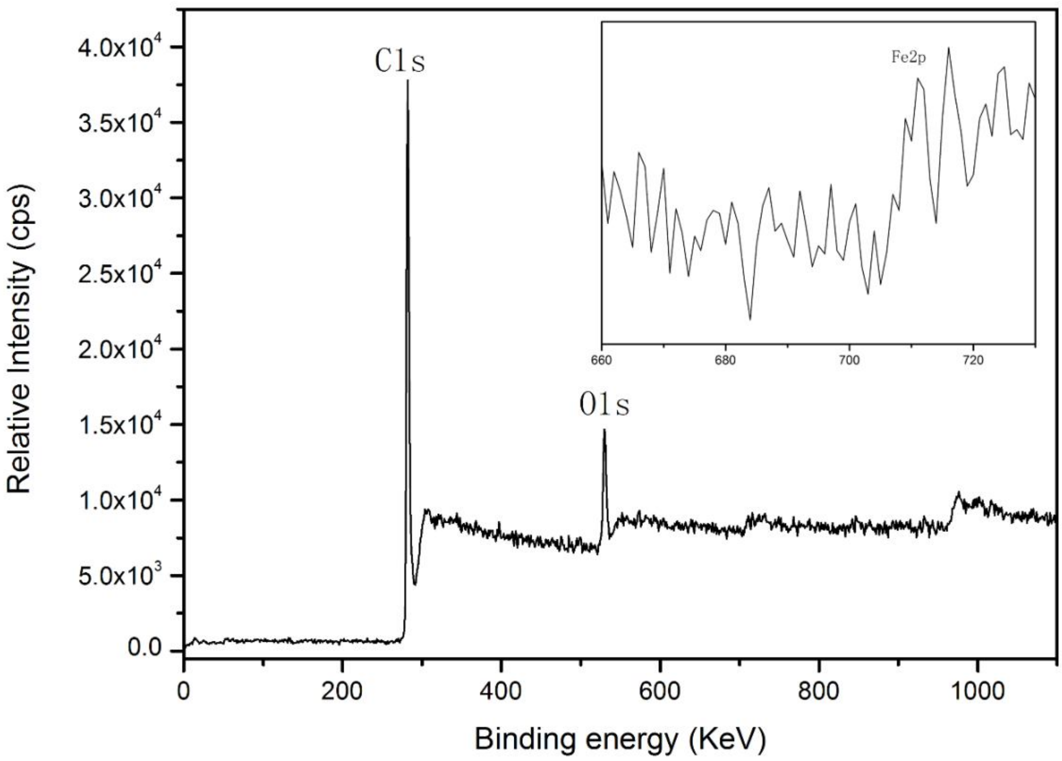

Figure S1. XPS spectroscopy of MCFNP surface. (Inset shows the enlarged area corresponded to the Fe element).

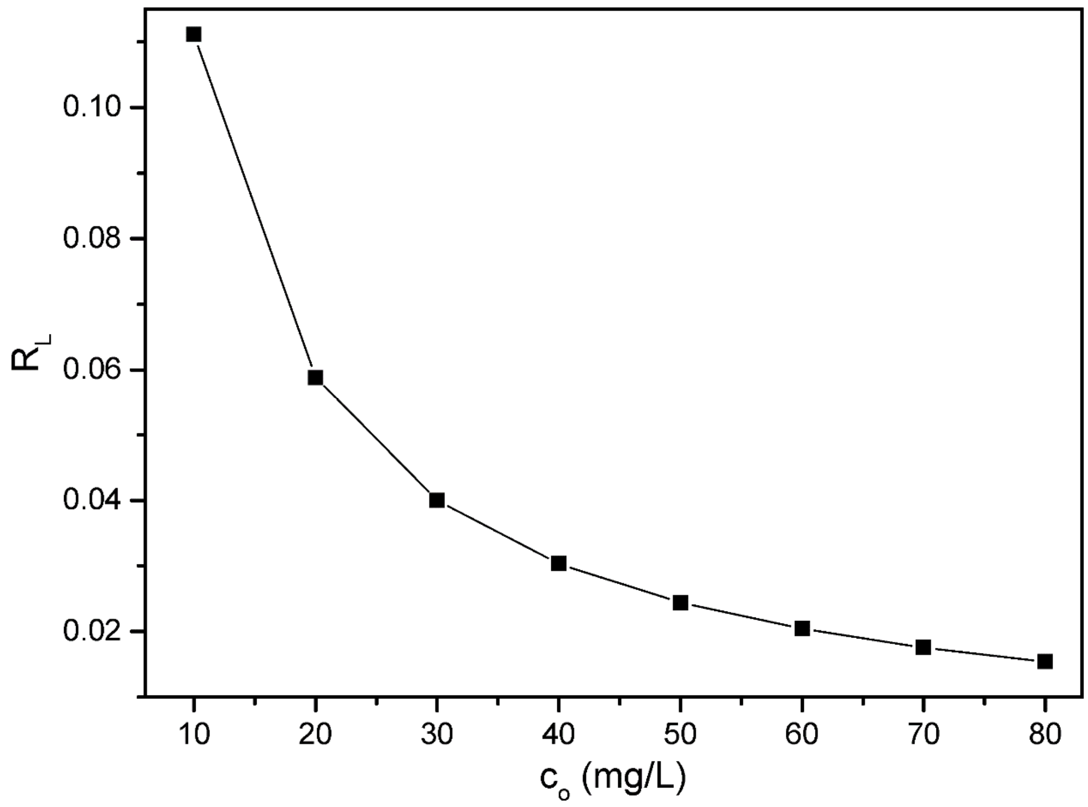

Figure S2. Separation factor R_L for adsorption of MB by MCFNP.

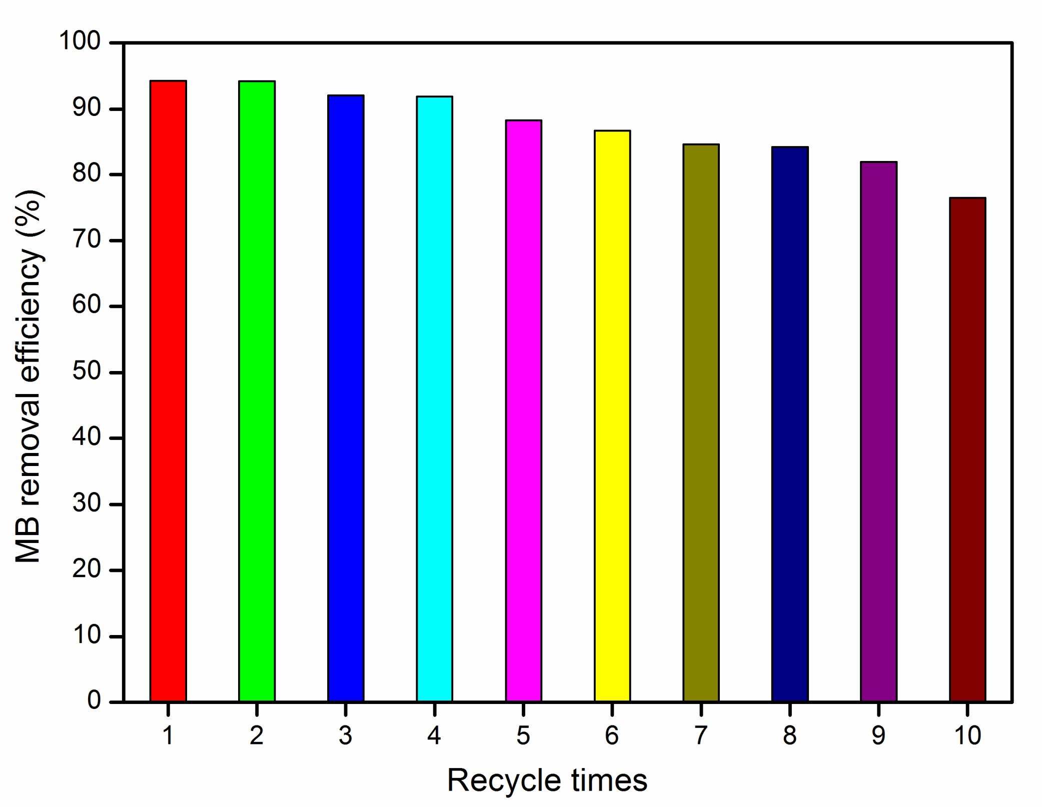

Figure S3. Removal efficiency of MB on MCFNP after different regeneration cycle times.

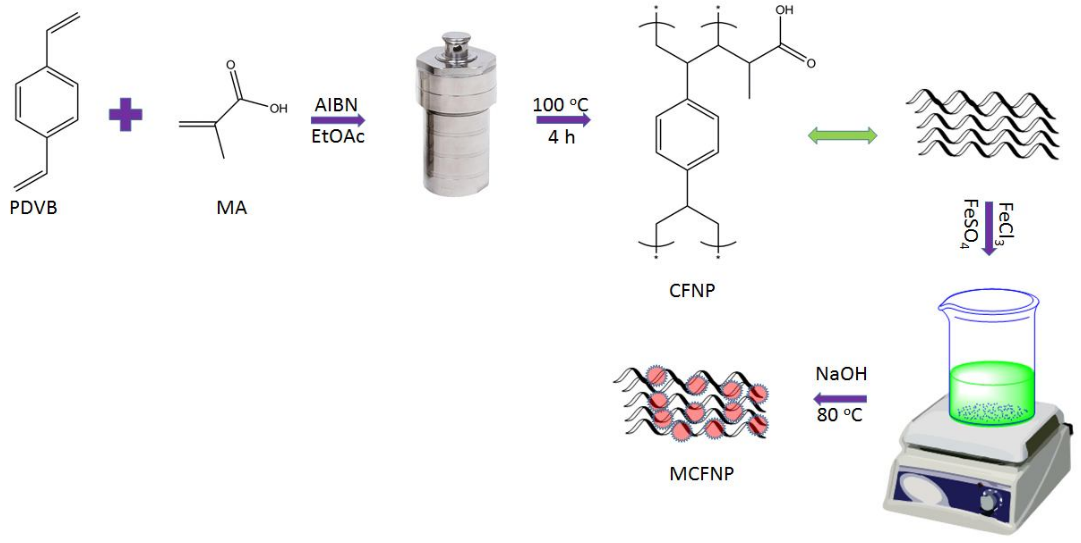

Figure S4. Illustration of MCFNP synthesis route.

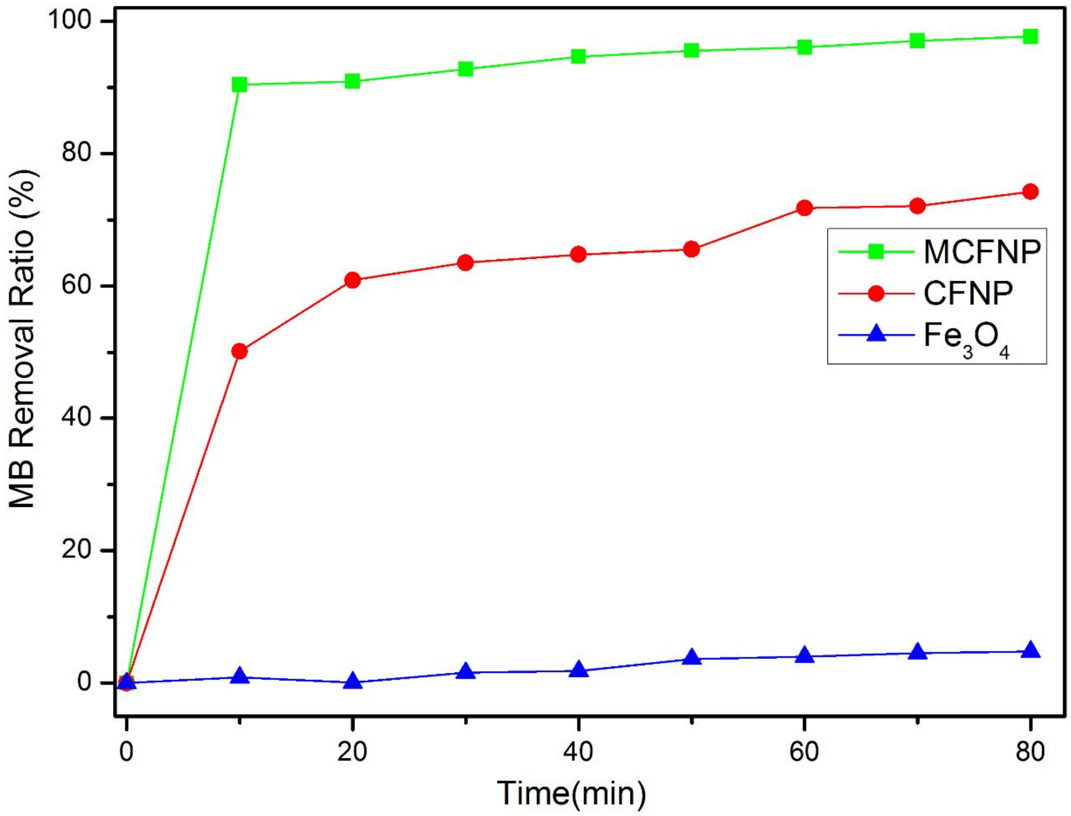

Figure S5. Adsorption performance of different adsorbents (MCFNP, CFNP and Fe_3O_4) for MB.
